# Benefits of public engagement in research and barriers to participation: a UK‐based survey of academic scientists and support staff including international respondents

**DOI:** 10.1111/imcb.70079

**Published:** 2026-01-09

**Authors:** Chioma M Ogbukagu, Matthias Eberl, Natalie Joseph‐Williams, Sarah Hatch, Jonathan M Tyrrell

**Affiliations:** ^1^ Institute of Life Science School of Medicine, Swansea University Swansea UK; ^2^ Division of Infection and Immunity School of Medicine, Cardiff University Cardiff UK; ^3^ Systems Immunity Research Institute Cardiff University Cardiff UK; ^4^ Division of Population Medicine School of Medicine, Cardiff University Cardiff UK; ^5^ Health and Care Research Wales Evidence Centre Cardiff UK; ^6^ Public Involvement and Engagement Team School of Medicine, Cardiff University Cardiff UK

**Keywords:** patient and public involvement, public engagement in research, responsible research and innovation, science communication

## Abstract

Public engagement is increasingly central to research, especially in biomedical fields, fostering dialogue between scientists and society, building trust and ensuring real‐world relevance. However, as scientific and clinical progress accelerates, the gap between researchers and the public continues to widen, underscoring the need for deeper, more meaningful engagement. Despite the acknowledged value of public engagement for both researchers and the public, we know relatively little about academics' views on opportunities and potential barriers to participation. Using questionnaires and interviews, this study captured insights from 99 researchers and professionals across academic disciplines, career stages and geographical and cultural contexts. Respondents consistently regarded public engagement as an important and rewarding aspect of research, teaching and institutional responsibilities, with the potential to enhance public understanding, acceptance and societal impact. However, enthusiasm was tempered by persistent barriers, including academic workloads, inadequate resources and support, and a lack of formal recognition within career progression. Respondents emphasized the need for systemic reforms to enable greater participation, including tailored training, sustained funding and institutional frameworks that acknowledge and reward engagement. Overall, the findings demonstrate that while motivation for public engagement is widespread, structural and systemic challenges limit its full potential. Addressing these barriers requires coordinated action from universities, funders and policymakers to establish and embed public engagement more consistently as an integral component of academic research and higher education.

## Graphical abstract

An examination of academics' participation in public engagement across disciplines, career stages and cultural contexts. Despite widespread enthusiasm and value for academic roles, systemic and structural barriers such as time, resources and recognition limit fuller participation. Surveys and interviews highlight the need for tailored training, sustained funding and institutional support.

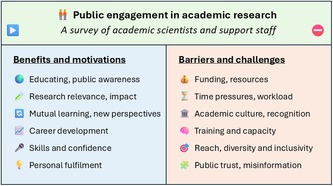

## INTRODUCTION

Public engagement comprises “the myriad of ways in which the activity and benefits of higher education and research can be shared with the public,” as defined by the UK's National Coordinating Centre for Public Engagement.^1^ It offers opportunities for a two‐way dialogue between scientists and the wider public, providing much‐needed context, building trust and ensuring real‐world relevance of research. Such pursuits have never been more pertinent—as scientific fields advance rapidly, there is a disconnect between researchers and the public, limiting both understanding and acceptance of the scientific process. Ethically sensitive issues such as embryonic stem cell research^2^ and vaccine uptake^3^, ^4^ are exemplars of this tension between personal beliefs, public perception and scientific literacy. However, despite the acknowledged benefits and increasing requirements for public engagement in academic practice, we know relatively little about researchers’ views and perceived barriers to participation. Understanding these is key to offer adequate support, training and solutions to the key challenges.

Recognizing the importance of public engagement in research is not new. In 1995, the UK Government convened the “Wolfendale Committee” of scientists and scientific journalists to develop a consensus on the public understanding of science, engineering and technology. The committee concluded that publicly funded scientists have a duty to explain their work, and recommended that public understanding should be embedded in research grants and that universities should prioritize communication skills training for researchers.^5^, ^6^ This trajectory culminated in the formalization of *Impact* within the UK Research Excellence Framework (REF), a nationwide review and evaluation of the research quality at UK Higher Education Institutions, which is crucial for prestige, core funding and the ability to attract staff and students. Since 2014, all research organizations in the United Kingdom have been required to submit *Impact Case Studies* as part of their REF returns, systematizing the evaluation of societal impact in higher education and leaning on the complementary role of patient and public involvement and engagement for academic research and innovation.^7^, ^8^

Public trust in science is key to the broader concept of responsible research and innovation.^9^ UK Research and Innovation (UKRI), the UK's public body that directs research and innovation funding, defines responsible research and innovation as a “process that seeks to promote creativity and opportunities for science and innovation that are socially desirable and undertaken in the public interest”;^10^ others describe it as “taking care of the future through collective stewardship of science and innovation in the present”.^11^ High‐quality public engagement, when combined with rigorous evaluation and targeted delivery, has the potential to strengthen this trust and advance the field by aligning scientific practice more closely with public needs and priorities.^12^ Major societal challenges including antimicrobial resistance, climate change and the COVID‐19 pandemic underscore the importance of effective strategies to engage, involve and educate the public in scientific research.^13^, ^14^

The growing importance of public engagement and the role it plays in addressing public health threats is acknowledged globally. For instance, both WHO^15^, ^16^ and the UK government^17^ identified public education and engagement as central to tackling the global spread of antimicrobial resistance. Similarly, UNESCO highlighted public engagement as essential to global responses to climate change because of the requirement not only of scientific and policy solutions but also of behavioural change, resilience and solidarity.^18^ Despite such clear mandates, translation into practice and meaningful change at the coalface of scientific research has been inconsistent, and cultural, attitudinal and structural barriers remain. Existing metrics of academic success and systems of recognition and peer esteem rarely prioritize public engagement, resulting in only limited integration into academic life.^1^, ^19^

Delivering public engagement to the quality needed to make an impact depends on significant participation and input from motivated researchers, and while public engagement‐focused professional support roles are increasingly common in research organizations,^20^ resource challenges persist. For example, our own “Superbugs” initiative required over 30 volunteers, from undergraduate students to senior academics, to deliver an interactive microbiology‐focused educational pop‐up shop in a public space.^21^ Complex engagement programs at institutional level can depend on even higher numbers of helpers and participants.^22^, ^23^ In this context, as important as understanding the needs and requirements of public stakeholders is understanding the motivations of scientists to engage.^1^, ^19^

Despite the widely accepted importance and benefit of public engagement to researchers and the public and academic institutions, there is a relative paucity of research on academics' attitudes and barriers to participation in public engagement. In Germany, Püttmann *et al*.^24^ identified safeguarding concerns in face‐to‐face engagement. UK‐based research found that many scientists saw communication with policymakers more as a priority than engaging and educating the public^25^; however, it is unclear whether this is still a widely held view. There is added confusion regarding whether and when ethical approval may be required for the conduct of public involvement and engagement activities,^26^, ^27^ potentially discouraging researchers from reaching out to the public.

Embedding public engagement meaningfully within higher education and research requires a deeper understanding of how academics perceive public engagement and what motivates or hinders their participation. Barriers may be structural or personal, and without addressing both, efforts to promote engagement risk being fragmented or ineffective. Such insights are essential for designing incentives, training and support systems that make engagement both feasible and rewarding. The present study aimed to address this knowledge gap by using questionnaires and semistructured interviews to examine perceptions of public engagement in academia and identify factors influencing participation among academics, researchers and science‐related stakeholders.

## RESULTS

### Data collection

#### Recruitment

The context of the present study and the corresponding questionnaire were primarily promoted via a dedicated page on the Superbugs website, a major public engagement platform run jointly by academics at Cardiff University and Swansea University (UK).^29^ Between August 1 and October 31, 2024, this study page was viewed 402 times, with an average time on page of 5:45 min (data not shown), demonstrating extensive engagement with the content. The button leading to the online questionnaire had 580 unique views and was clicked 183 times, corresponding to a conversion rate of 30.9% (data not shown). These 183 clicks ultimately translated into the successful submission of 99 completed questionnaires through Microsoft Forms between August 5 and October 19, 2024.

#### Questionnaire respondent cohort

Table 1 illustrates the diversity of respondents in terms of age, gender, career stage, academic discipline and geography/origin. Most responses were from people currently based in the United Kingdom, including individuals of EU origin (Ireland, Poland, Italy, Cyprus and Romania) and from outside Europe (Nigeria, Ghana, Mexico and Australia). International responses came from Austria, Canada, the United States, Turkey and United Arab Emirates (data not shown). Females comprised 75.8% (75/99) of the cohort. Respondents came from across the spectrum of academic positions and career stages and included Early Career Researchers (ECRs): postgraduate students or postdoctoral researchers, Lecturers/Senior Lecturers (and those at a comparative level of independence), Associate Professors/Professors, Professional Services staff and nonacademic support roles, and Alternative Careers such as scientists and miscellaneous roles in other sectors (Table 1). Of note, nearly half of respondents classified as “Professional Services” consisted of support staff directly involved in the delivery of public engagement such as institutional engagement leads, managers and advisors (data not shown). Areas of expertise and academic disciplines were heavily orientated toward health‐related subjects, including Biomedical Science (53.5%), Medicine/Healthcare (40.4%) and Psychology/Social Care (19.2%) (Table 1). Being able to select more than one area of expertise, 39.4% of respondents identified themselves as multi‐disciplinary (data not shown).

**Table 1:** Key demographics of respondents.

	Number (*n*)	Percentage of cohort (%)
Age (years)		
<21	0	0
21–30	20	20.2
31–40	29	29.3
41–50	32	32.3
>50	17	17.2
Prefer not to say	1	1.0
Gender		
Male	20	20.2
Female	75	75.8
Nonbinary	1	1.0
Prefer not to say	3	3.0
Religion		
Christian	42	42.4
Muslim	4	4.0
Hindu	1	1.0
None	46	46.5
Prefer not to say	6	6.1
Geography/origin		
UK resident (UK origin)	58	58.6
UK resident (EU origin)	10	10.1
UK resident (non‐EU origin)	21	21.2
Non‐UK resident	10	10.1
Language of education		
English	92	92.9
English in combination with second language	3	3.0
Language other than English	4	4.0
Area of expertise^a^		
Biomedical Science^b^	53	53.5
Medicine/Healthcare	40	40.4
Psychology/Social Care	19	19.2
Biosciences/Natural Sciences	20	20.2
Mathematics/Physics	13	13.1
Other	8	8.1
Current career position		
Early career researcher	22	22.2
Lecturer/Senior Lecturer	19	19.2
Associate Professor/Professor	16	16.2
Professional services	24	24.2
Alternative careers^c^	18	18.2
Years of experience in academia		
≤5	28	28.3
6–10	21	21.2
11–15	18	18.2
>15	32	32.3
Years of experience in public engagement		
≤5	38	38.4
6–10	26	26.3
11–15	10	10.1
>15	25	25.3

^a^ Respondents were able to select more than one area of expertise to best capture the scope of subject matter covered in the present cohort.

^b^ Microbiology, Immunology, Cell Pathology and other laboratory‐based biomedical science subjects.

^c^ Nonacademic scientists and miscellaneous careers.

#### Interview subcohort

Individuals selected for in‐depth interviews were representative of the wider respondent diversity, comprising four females and three males aged 21–50 years. Interviewees were from multiple disciplines spanning medical sciences, psychology, linguistics, engineering and geosciences, and included ECRs, Lecturers/Senior Lecturers and Professional Services staff, with ≤5 years to 15 years of experience in public engagement.

### Survey and interview results

Our findings are organized around the topics explored in the questionnaire and the key themes emerging from the interviews: participation in public engagement, types of public engagement, training, motivations and attitudes, benefits of public engagement, barriers to public engagement, key challenges and limitations, and effective strategies. Where a theme also emerged from the interviews, we provide exemplar quotes.

#### Participation in public engagement

**Figure 1:**
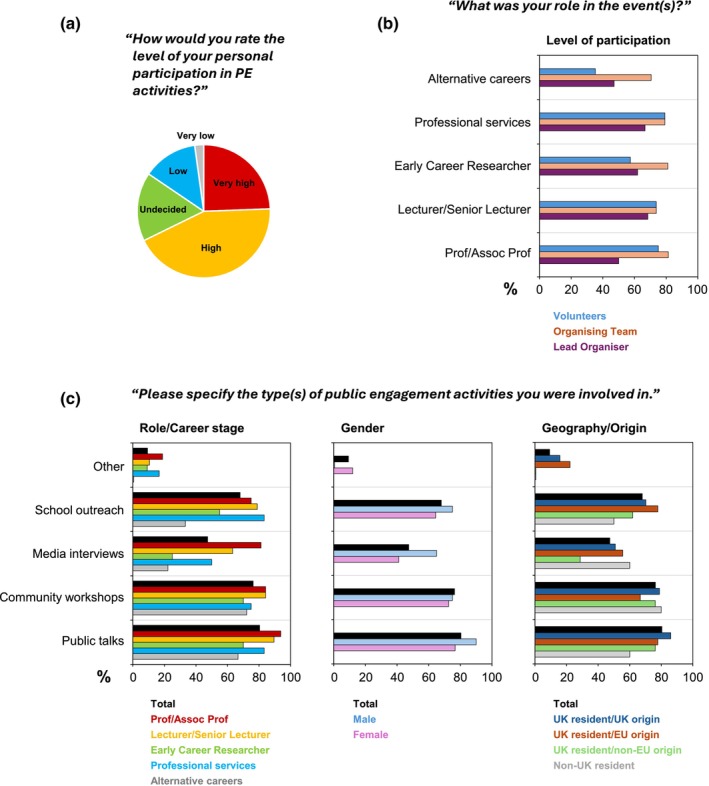
Contributions to public engagement activities respondents were involved in, shown as percentages within diverse subgroups in response to the questions shown, according to the respondents’ professional role and career stage (left), gender (middle) and geographic location and origin (right). Alternative careers: Nonacademic scientists and those of other career pathways. The breakdown according to gender excludes three individuals who selected “Prefer not to say” as an option, and one individual who identified as nonbinary. **(a)** Q29: “How would you rate the level of your personal participation in public engagement activities?” **(b)** Q13: “What was your role in the event(s)?” **(c)** Q11: “Have you participated in any public engagement activities?”—Q12: “If yes to Q11, please specify the type(s) of public engagement activities you were involved in.”

Most respondents had prior experience of public engagement activities, ranging from ≤5 years to >15 years of experience (Table 1). More than two thirds of respondents (68.5%) stated that their level of personal participation in public engagement activities had been “Very high” or “High” (Figure 1a).

Age seemingly had an impact on the nature of public engagement participation, with fewer respondents >50 years old indicating they participated in a volunteer role only (41.2%), in agreement with a similarly low rate for Associate Professors/Professors (50.0%) (data not shown). In contrast, the likelihood to take a leadership role in public engagement was highest in those >50 years old (82.3%) (data not shown). When analyzed according to career stage, those in Professional Services (79.2%), Lecturers/Senior Lecturers (73.7%) and Associate Professors/Professors (75.0%) were far more likely to take leadership roles, as opposed to ECRs (57.1%) (Figure 1b).

Females were comparatively more likely to volunteer and take on leadership roles in public engagement compared to males (64.0% vs. 55.0%, respectively, in each case). Contrastingly, 85.0% of males indicated they had participated as part of an organizing team, compared to 73.3% of females (data not shown).

#### Types of public engagement

The most common types of public engagement activity respondents had participated in were “Public talks or lectures” (80.4%), followed by “Community workshops or seminars” (76.3%) and “School outreach events” (68%) (Figure 1c). Still, almost half of respondents had experience with “Media interviews and/or articles” (47.7%). Other contributions ranged from community outreach events and festivals to science‐art projects, collaborations with theatres and film makers, and events targeted at patient recruitment for research and clinical trial activities. Rates of participation in all forms of public engagement were higher in males than in females. ECRs were far less likely to contribute to media interviews/articles (25.0%) than those in the more advanced positions of Lecturer/Senior Lecturer (63.2%) and Associate Professor/Professor (81.3%) (Figure 1c).

Of note, almost all respondents (95/97, 97.9%) had participated in public engagement activities delivered in English. However, one in five respondents listed additional experience in delivering at least some aspects of their public engagement activities in languages including Welsh, French, Spanish, German, Finnish, Turkish, Arabic, Urdu, Igbo, Yoruba, Twi and British Sign Language (data not shown), illustrating the multicultural background of the cohort and the readiness to engage diverse communities.

#### Motivations and attitudes

**Figure 2:**
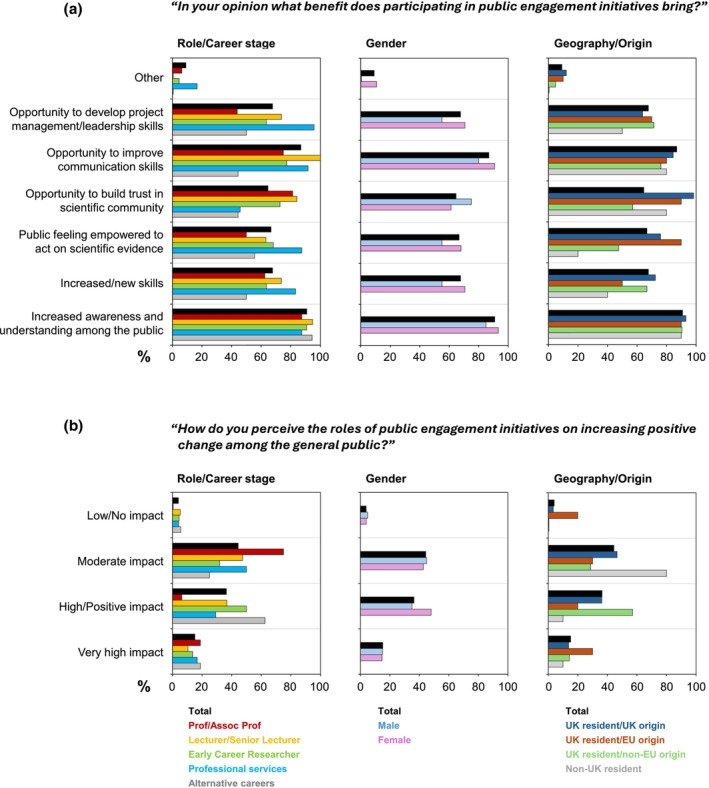
Motivations and value of participating in public engagement within diverse subgroups in response to the questions shown. **(a)** Q18: “In your opinion what benefit does participating in public engagement initiatives bring?” **(b)** Q19: “How do you perceive the roles of public engagement initiatives on increasing positive change among the general public?”

Respondents saw multifaceted motivations to participation in public engagement. Overall, they identified “Increased awareness/understanding in the public” (90.9%) and “Improved communication skills” (86.9%) as the primary benefits of public engagement activities. While comparatively lower, “Increased skill set” (67.7%), “Improved management/leadership skills” (67.7%), “Empowering the public to act on scientific evidence” (64.6%) and “Building trust in the scientific community” (64.6%) were still important benefits (Figure 2a). This majority view that public engagement plays a generally beneficial role was consistent with the fact that almost three quarters (74.7%) reported positive outcomes resulting from public engagement initiatives they had participated in. Only three individuals said that they had not experienced any positive outcomes—two of whom had in fact never undertaken any public engagement (data not shown).

While most demographic groups broadly shared similar views overall, two differences stood out. First, although all agreed that raising awareness and understanding is a key benefit of public engagement, a much larger proportion of senior academics and ECRs saw public engagement as a way to build trust in the scientific community, compared to professional services staff and those on other career paths (Figure 2a). Second, geographic location and origin influenced whether respondents viewed public engagement as a means of empowering the public to act on scientific evidence. This was highlighted as a public engagement benefit by most UK residents of UK or EU origins but was less frequently selected by UK residents of non‐EU origins, and rarely by respondents outside the United Kingdom, suggesting potential cultural differences (Figure 2a).

#### Benefits of public engagement

**Figure 3:**
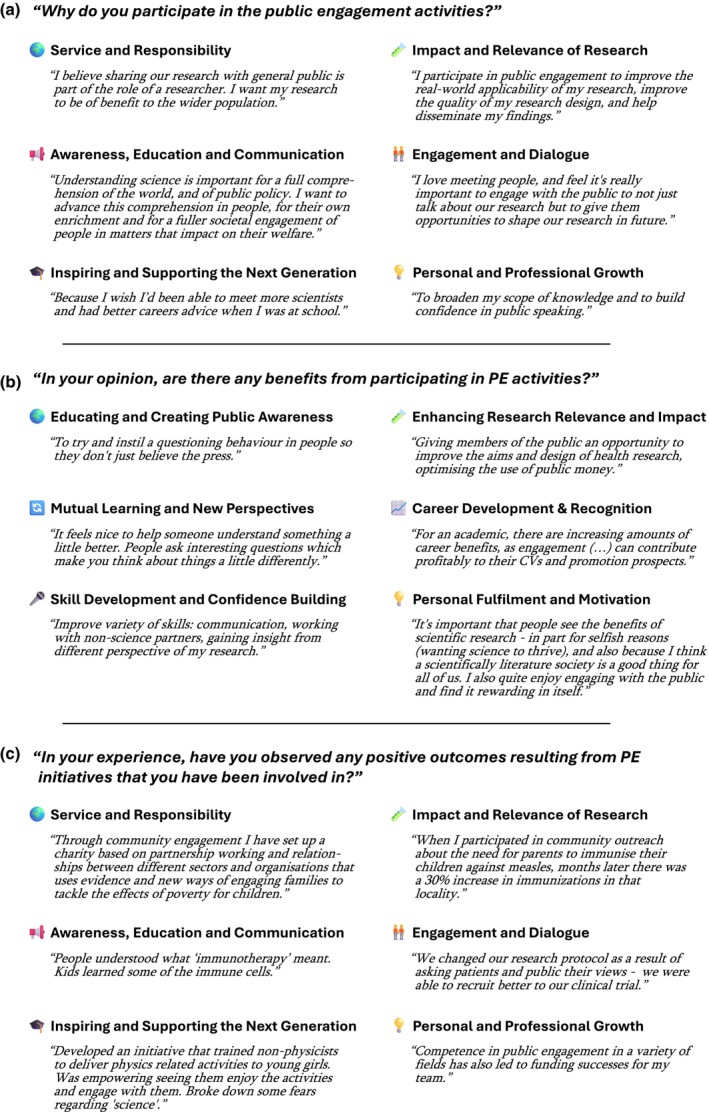
Benefits and positive outcomes of public engagement, as identified from open‐ended responses to the three questions shown, alongside illustrative quotes by selected respondents. **(a)** Q14: “Why do you participate in public engagement activities?” **(b)** Q15: “In your opinion are there any benefits from participating in the public engagement activities?”—Q16: “If Yes to Q15, kindly state the benefits.” **(c)** Q21: “In your experience, have you observed any positive outcomes resulting from public engagement initiatives that you have been involved in?”—Q22: “If Yes to Q21, kindly explain your experience.”

Coding of open‐ended questionnaire responses identified “Service and Responsibility”, “Impact and Relevance”, “Awareness, Education and Communication”, “Engagement and Dialogue”, “Inspiring and Supporting the Next Generation” and “Personal and Professional Growth” as key benefits motivating respondents’ participation in public engagement (Figure 3a; Supplementary table 3). These views were consistent across academic disciplines. The dominance of themes such as responsibility, awareness and education mirrored the fact that half of all respondents perceived public engagement as having a “High” or “Very high” level of impact on increasing positive change in the public (Figure 2b).

Major benefits from participating in public engagement activities could be divided into societal benefits (“Educating and Creating Public Awareness”, “Enhancing Research Relevance and Impact”, “Mutual Learning and New Perspectives”) and personal/professional benefits (“Career Development and Recognition”, “Skill Development and Confidence Building”, “Personal Fulfilment and Motivation”) (Figure 3b; Supplemental table 4). Direct examples for positive outcomes resulting from public engagement initiatives respondents had participated in spanned all six key motivation areas (Figure 3c; Supplemental table 5).

#### Training

The perceived importance of training in public engagement is stressed by a free text quote from the questionnaire.

“Most academics don't value/prioritise public engagement, which means most aren't trained in it (including students). The result is a lot of ineffective and top‐down/deficit‐model ‘communication’ that doesn't land well (at best) and is harmful at worst.” SP65 (female Associate Prof./Professor, non‐UK resident)

Only 54.5% of respondents had undergone any public engagement‐related training (data not shown). The frequency of this training varied greatly; of those who had received training, the majority indicated that this was on an annual (53.7%) or monthly basis (25.4%) (data not shown). Employees in professional academic services were the most likely to receive regular training, with 5.9% receiving training weekly and 29.4% monthly. In comparison, training was less common among academic staff: none of the Associate Professors/Professors reported weekly or monthly training and only 16.7% of Lecturers/Senior Lecturers had received training on a monthly basis (data not shown).

**Figure 4:**
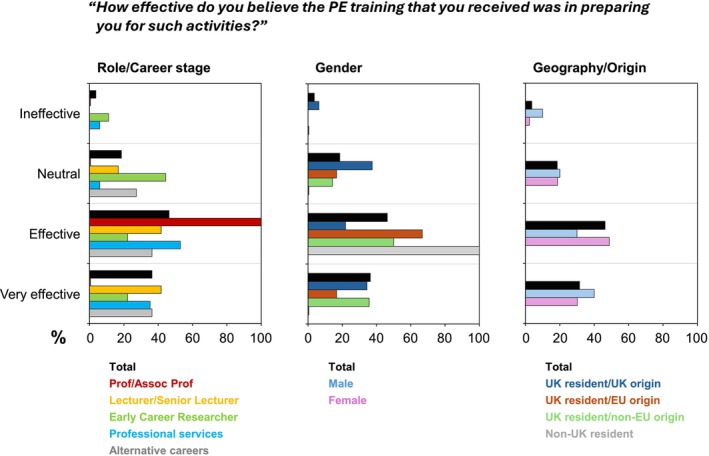
Effectiveness of public engagement training, shown as percentages within diverse subgroups in response to question Q32: “How effective do you believe the public engagement training you have received was in preparing you for such activities?”

Encouragingly, perceptions of the quality, impact and value of the training were strongly positive. Among those who had received training, only two individuals felt that the training had been “Ineffective” in preparing them for public engagement activities. ECRs were particularly uncertain about the value of public engagement training, with almost half selecting a Neutral response (Figure 4). In contrast, most Lecturers/Senior Lecturers and all Associate Professors/Professors rated the training as “Effective” or “Very Effective”. With regard to geography and cultural background, more respondents with EU or non‐EU origin rated the training as “Effective” or “Very Effective” than respondents with UK origin (Figure 4).

#### Barriers to public engagement

Overall, 85.9% of questionnaire respondents stated that they had faced barriers to participation in public engagement, including one respondent who indicated they had not previously done any public engagement (data not shown). The overall struggles academics face when attempting to participate in public engagement activities are perhaps best illustrated by two free text quotes from the questionnaires.

“It's not necessarily valued—universities make a song and dance about it when it happens but they make very little effort to actually establish or support it. As academics we rarely have the time to do it well.” SP18 (male Associate Prof./Professor, UK)

“Senior academics/group leaders not seeing the value, seeing it as a waste of time and therefore discouraging/not supporting students/postdocs in participating, departments not providing supported opportunities for junior students to learn and engage, unsuitable facilities, access issues, lack of provision for disabled people.” SP77 (female ECR, UK)

**Figure 5:**
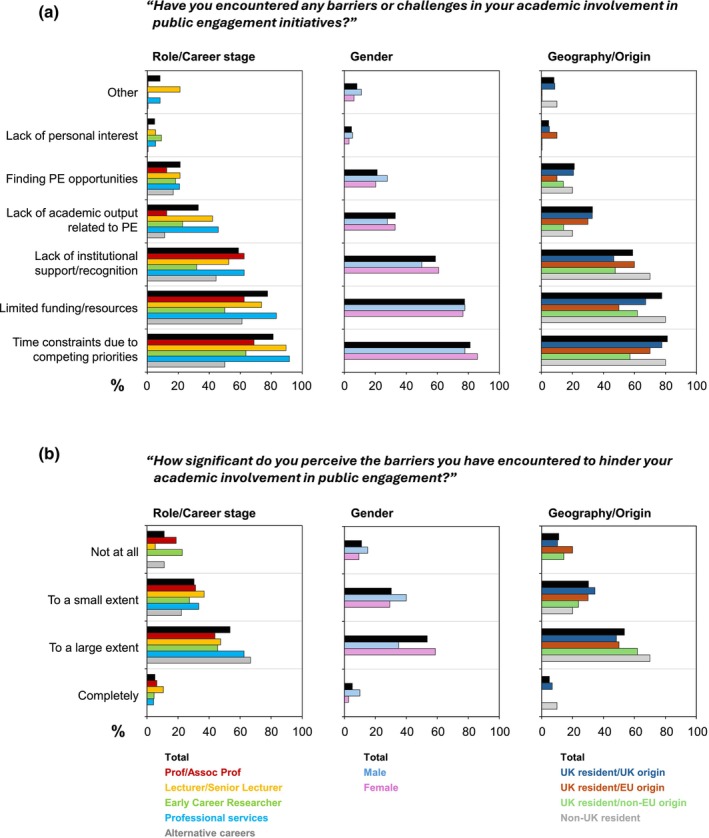
Barriers to participation in public engagement activities and their perceived significance within diverse subgroups in response to the questions shown. **(a)** Q34: “Have you encountered any barriers or challenges in your academic involvement in public engagement initiatives?”—Q35: “If Yes to Q34, please select the primary barrier(s) you have encountered.” **(b)** Q36: “How significant do you perceive the barriers you have encountered to hinder your academic involvement in public engagement?”

Figure 5a highlights the main barriers encountered by the questionnaire respondents, chosen from a range of predefined answer options. The most frequently reported challenges were “Time Constraints due to competing priorities” (81.2%), “Limited Funding/Resources” (77.6%) and “Lack of Institutional Support/Recognition” (58.8%). These obstacles were consistently noted across all career stages. In contrast, “Lack of personal interest” was mentioned by only 4.7%. Respondents also raised additional issues, including universities prioritizing (“not unreasonably,” as pointed out by one respondent) student recruitment over public engagement, difficulties in finding effective ways to reach the public or patients, and reluctance of colleagues to participate. Notably, “Lack of academic output” was cited by over 30% of respondents as a perceived barrier, especially by those in Professional Services roles and at Lecturer/Senior Lecturer level.

Only 34.3% of respondents indicated they thought there was a “Large extent of support” for public engagement participation at their host institution (data not shown). There was no statistical difference between the rates at which each barrier to public engagement participation was identified by males and females. The largest gender difference came with “Lack of Institutional Support/Recognition”, with >10% more females experiencing this than males. Of interest, structural or systemic barriers (“Time constraints due to competing priorities”, “Limited funding resources” and “Lack of institutional support/recognition”) were consistently highest in those of non‐UK residency, whereas “Lack of academic output” was less of an issue in that group.

**Figure 6:**
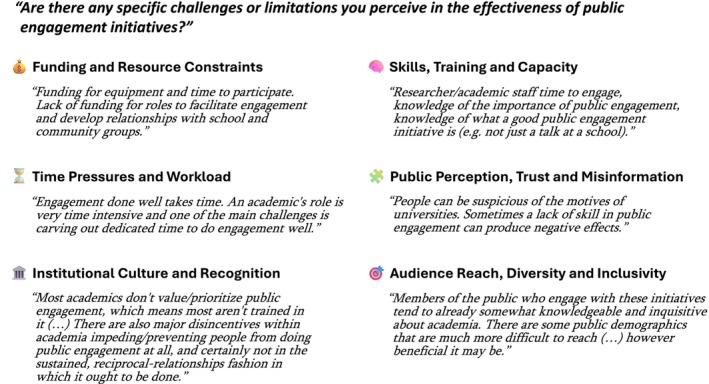
Challenges or limitations of public engagement, as identified from open‐ended responses to the question shown, alongside illustrative quotes in response to question Q24: “Are there any specific challenges or limitations you perceive in the effectiveness of public engagement activities?”—Q25: “If Yes to Q24, kindly explain the challenges experienced.”

Open‐ended reflections on the effectiveness of public engagement initiatives allowed us to identify “Funding and Resource Constraints”, “Time Pressures and Workload”, “Institutional Culture and Recognition”, “Skills, Training and Capacity”, “Public Perception, Trust and Misinformation” and “Audience Reach, Diversity and Inclusivity” as perceived challenges and limitations (Figure 6; Supplemental table 6).

In‐depth interviews of seven respondents to the original questionnaire confirmed the existence of systemic barriers, especially concerning limited resources and time, unsatisfactory recognition in terms of career progression and lack of effective public engagement training. Notably, the lack of academic outputs as emerging from the questionnaires was not a dominant concern in the interview subcohort.

“There is no (…) recognition if you do really good public engagement. It's just something you're expected to do.” SP70 (female Lecturer/Senior Lecturer, UK)

“It's time really. It takes planning, meetings, coordination. And when you're balancing teaching and research, it feels like extra work.” SP40 (female Professional Services, non‐UK resident)

The barriers identified in the present study carry clear significance. Just over half of respondents indicated that the perceived barriers hindered their public engagement efforts “To a large extent,” and an additional third noting an effect “To a small extent” (Figure 5b).

#### Solutions to overcome perceived barriers

When asked in the questionnaire to identify strategies by which academic institutions may overcome perceived barriers to participating in public engagement, the most commonly suggested solutions included “Flexibility in workload to accommodate public engagement activities”, “Increased institutional support” and “Dedicated funding for public engagement activities”, highlighting a clear correspondence with the barriers initially identified (data not shown). As such, those answers directly reflected the challenges and limitations highlighted earlier (Figure 5b).

Additional insight was gained from the interviews, in which the respondents stressed the need for sustained and structural support in academic institutions, rather than occasional and intermittent goodwill gestures.

“Just making it visible—awards, recognition, career pathways. If you give it equal weight to teaching and research, more people will do it properly.” SP70 (female Lecturer/Senior Lecturer, UK)

“More targeted training would help—not just generic ‘how to talk to the public’ but sessions on working with children, or on engaging communities that aren't usually reached.” SP83 (male Lecturer/Senior Lecturer, UK)

#### Effective approaches to impactful public engagement

Above all, interviewees were enthusiastic about undertaking public engagement and enjoyed having the opportunity to pass on their views of best practices in public engagement. In this respect, they were keen to emphasize the critical role of delivery strategy in shaping public engagement projects/events, as well as the overall impact of the work. They also highlighted the importance of tailoring delivery methods to suit specific stakeholder groups. A clear distinction was drawn between public engagement activities aimed at fostering awareness behaviours (such as short, snapshot‐style initiatives) and those aimed at driving complex behavioural change (requiring long‐term collaborative/co‐produced engagement), including the significance of securing sufficient and sustainable funding for the latter.

“Approach depends on the audience. For schools, we'll use games and experiments, for community groups it's more discussion based (…) Storytelling is a big part of our delivery—people remember stories more than statistics.” SP37 (female ECR, UK resident with EU origin)

“If you just want awareness, a one‐off event or festival can work. But if you want real behavior change, you need long‐term sustained work with the same group.” SP83 (male Lecturer/Senior Lecturer, UK)

“We rarely get working‐class families or people from ethnic minorities at events. It tends to be middle‐class educated people.” SP51 (male ECR, UK)

A key takeaway from the interviews was that impact evaluation of public engagement remains underdeveloped and often superficial. In particular, capturing long‐term behavioural change was seen as a considerable challenge. Interviewees acknowledged that evaluation is frequently treated as an afterthought, largely owing to time and funding constraints.

“We're pushed to show evidence of impact but no one really agrees with what impact really means.” SP61 (female Professional Services, UK)

“There's pressure to show measurable impact but the reality is that most of it is qualitative and very difficult to evidence.” SP70 (female Lecturer/Senior Lecturer, UK)

## DISCUSSION

This study explored academics' experiences, barriers and facilitators to participating in public engagement. Our findings describe the perceptions and attitudes of broadly motivated academics from diverse backgrounds, across a range of career pathways, who viewed public engagement as a vital and impactful aspect of their research, teaching and support roles. As the separation between science communication, outreach and public engagement can be quite fluid in practice, and the terms are often used interchangeably, especially across disciplines and countries,^30^ the present questionnaire aimed to capture the full scope of participation in public‐facing activities. Importantly, our findings go beyond previous studies^31^, ^32^, ^33^ by adding further nuance with regard to different types of engagement, the degree of participation versus leadership roles, the inclusion of professional service staff and alternative careers, and the pivotal requirement to evidence the impact of public engagement activities.

### Varying public engagement experiences

The nature of public engagement participation reported by our respondents was very varied, including public talks and lectures, school and community engagement, art‐based projects and media interviews or articles. As academic careers progress, our findings suggest a shift in the types of activities, moving from two‐way dialogue and mutual benefit to one‐way science communication activities such as media interviews and, perhaps unsurprisingly, an increase in leadership roles. This transition is underpinned by a positive view on the impact that training had on subsequent public engagement activities. While male respondents reported diversity of participation across all public engagement activities listed, slightly more females than males assumed a leadership role. ECRs were less likely to give media interviews, possibly due to a relative lack of confidence, experience and opportunity, even though they increasingly see public engagement as part of their role.^34^, ^35^

Our sample was 75% female. This may simply indicate a somewhat stronger inclination of females to complete surveys like the present one but is in accordance with the reported overrepresentation of women in public engagement,^36^, ^37^ which may align more closely to female societal, relational and communication styles.^38^ In our study, we found that more males reported taking part in media interviews. In relation to our observation that media interviews are typically undertaken by more senior academics, we know that senior academic positions continue to be disproportionately occupied by men.^39^ A survey in Italy found a gender gap in media‐related engagement but not in community‐based activities, pointing to the nuanced nature of gender differences in public engagement participation.^40^ This is supported by our study, where both genders were equally likely to participate in school outreach and community workshops. While present imbalances may be narrowing,^41^ the gender dynamics surrounding public engagement are still not fully understood and warrant further investigation, exploring key issues such as unequal workloads, gatekeeping, stereotypes about authority in science, imposter phenomenon and gendered criticism.^42^, ^43^

Our study identified a striking cultural difference in the perception of societal benefits of public engagement. Regardless of their gender, career stage and geographical location or origin, respondents equally subscribed to the view that public engagement boosts public awareness and understanding. However, the view that public engagement helps the public feel empowered to act on scientific evidence was dominant in UK residents of UK and EU origin but almost nonexistent in respondents outside the United Kingdom. While our study sample size was too small to look into possible underlying factors, this may point toward a difference in the cultural interpretation of public engagement. In the United Kingdom, there is a strong emphasis on patient and public involvement in research, extending the concept of merely engaging members of the public *about* research and instead, wherever feasible, involving them *in* the actual research, from study design to study conduct, and coproducing outputs.^44^, ^45^, ^46^ This level of empowerment in decision‐making processes is spearheaded by the United Kingdom (not the least because this is often a mandatory requirement expected by UK funders) and may not be as prevalent in other countries.^47^, ^48^, ^49^

### Overcoming barriers to public engagement

Key public engagement benefits identified in this study included both societal and personal/professional benefits. However, despite widespread enthusiasm for public engagement, respondents highlighted key structural and environmental barriers such as time constraints due to competing demands, limited funding or resources, and lack of institutional and professional recognition. Fostering trust in science depends on many actors, including researchers, educators, policymakers and the media, and requires coordinated efforts across institutions and society.^50^ While it is broadly acknowledged that public engagement serves as a valuable tool to make science and research more accessible, relevant and socially meaningful^51^—a view echoed by many respondents in this study—our findings confirm the persistence of deeply rooted obstacles that limit the potential reach of such impact.

Unsurprisingly, with the current “high effort–little reward” model of public engagement, sustained participation is often challenging in academic settings with pressures on prioritising outputs, research income and teaching. To mitigate these challenges, there was consensus across our sample around the need for removing at least some of these barriers and offering improved training, long‐term funding and systemic support to enable meaningful, inclusive and impactful public engagement. Notably, females and respondents from outside Europe were more likely to encounter barriers to public engagement than males.

### Potential solutions and drivers for change

#### Recognition

A persistent challenge for academic scientists, and highlighted throughout our cohort, is the lack of formal recognition of public engagement.^52^ Major funders often stipulate that an explicit plan for patient and public involvement and engagement is required for grant applications to score highly or be considered at all. However, even when embedded in the research, these public‐facing activities rarely contribute directly to the core academic currencies of papers and intellectual property. In the present study, the lack of academic outputs as a significant barrier was most apparent for those at the stage of Lecturer/Senior Lecturer, where they are paramount to reach probationary targets, professional development milestones and, ultimately, career progression.

The UK government's plans for *Engagement and Impact* to account for 25% of the overall REF assessment in 2029 does, on paper, provide an additional incentive. Yet, impact cases in biomedical and health sciences typically emphasize impact on policy, industry or clinical practice, rather than local grassroots initiatives like school outreach, science festivals or partnerships with community groups. Without recognition beyond traditional academic measures, public engagement risks remaining a secondary “add‐on” activity, carried out by committed individuals despite structural disincentives, rather than a genuinely valued part of the research process.^32^, ^52^ The increasing number of awards for science communication and public engagement at university level and by learned societies is a welcome step in this direction.^53^, ^54^

#### Structural support

Incremental improvements in the scope and size of public engagement‐related funding are not yet adequate to provide true incentives for substantial academic participation. Existing models fail to invest in sustainable public engagement infrastructure and are instead geared toward limited ad hoc projects, which fail to maximize expertise, avoid duplication and progress along pathways to impact. It is encouraging to see in our study the breadth of professional services staff dedicated to supporting public engagement activities at their institutions. However, these roles are often ill‐defined, understaffed and poorly networked,^1^ despite at least two decades of recommendations to improve administrative support and formally recognize outreach in academic evaluations to increase participation.^36^

#### Training

Structural support for public engagement must go beyond provision of resources. Our findings reveal a clear lack of adequate training as a major barrier to participation in public engagement. This is in agreement with earlier studies suggesting a need for sustained action by education systems and research institutions to embed science communication training and infrastructure directly into doctoral training programs.^34^, ^55^ The effectiveness of accessible science communication training across career stages to improve scientists' engagement with nonexpert audiences and boost their confidence is well established.^56^ This notion was confirmed in the present study where the overwhelming majority of respondents found the public engagement training they had received effective or very effective. A bottom‐up approach to training, introducing the importance, approaches, and philosophies of public engagement at an undergraduate and Master level of academia may act to breed an intrinsic valuation of the field in maturing scientists.^57^, ^58^

#### Impact evaluation

Public engagement is built on diversity, variety and creativity—this is what often initially captures the imagination and attention of the public we wish to engage with,^59^ but this may be counterintuitive to rigorous and reproducible methodology and evaluation,^60^ as highlighted by many interviewees in the present study. The increasing dissemination of public engagement‐focused research in scientific journals undoubtedly provides a positive feedback to share best practices both for delivery and for evaluation, and, at the same time, produces outputs to improve academic recognition of public engagement itself.^61^, ^62^ It is hoped that this development will help alleviate at least some of the systemic barriers to public engagement participation identified in our study.

### Strengths and weaknesses

We are confident that our sample covered a sufficiently diverse range of experience and expertise for an in‐depth analysis of the views of distinct subgroups on perceived benefits and barriers to public engagement, with regard to geography, career stage, gender and, to a lesser extent, academic discipline. An average completion time of 15:21 min for our questionnaire indicated genuine passion and interest in the study topic, which was corroborated by the shared appreciation of the value of public engagement underpinning the responses. However, we recognize the inherent bias in our approach in that a comprehensive understanding of the factors contributing to hesitancy or resistance toward public engagement requires the inclusion of more voices that do not align with the prevailing consensus in this study. Reaching out to academics who may be uncertain about, or even opposed to, public engagement—and, as a matter of fact, unwilling or unable to complete online questionnaires—would be essential for a balanced and rigorous exploration of this important topic. As such, the current study format unfortunately does not allow for an equitable dialogue across the divide of opinions on public engagement. Further work will act to provide a larger and more robust sample.

Overall, our study confirms that enthusiasm for public engagement in scientific research is widespread across academics but constrained by systemic and structural barriers.^30^, ^31^, ^32^, ^33^, ^34^, ^35^, ^36^, ^37^, ^52^, ^55^ Despite the well‐established benefits for both academics and stakeholders and the importance for strengthening science‐society relations, the cultural change toward fully embracing public engagement alongside the traditional roles of research and teaching is frustratingly slow.^19^ Our findings underline calls for higher education and research institutions as well as funders to embed public engagement more firmly into career recognition systems, provide dedicated training and resources, and support evaluation methods to assess outcomes and impact. Without such reforms, public engagement risks remaining an undervalued aspiration, rather than an integral part of academic science. Our study, and the associated work highlighted within, is testimony to a motivated community that would benefit from such reforms.

## METHODS

### Ethical approval

Permission for this study was obtained from Swansea University Ethical Review Board (Approval Number 2‐2024‐9829‐9824). All work and data protection complied with institutional protocols and ethical standards.

### Questionnaire

To collect views from a representative sample of the scientific community, we developed a comprehensive structured questionnaire in collaboration and consultation with public engagement leaders working in academic and professional services, consisting of 11 open‐ended questions, 7 binary Yes/No questions and 20 multiple‐choice questions (Supplementary table 1). Broadly, questions covered basic demographics, participation in public engagement, academic valuation of public engagement and perceived barriers to participation. The questionnaire was created in Microsoft Forms to facilitate electronic distribution and data analysis. Recruitment of respondents was active throughout August to October 2024 and was achieved via (1) a project‐specific page on the Superbugs website (https://www.superbugs.online/superblog/academic‐attitudes); (2) promotion on social media (@JTyrrell_Micro, @EberlLab, @CUSuperbugs) (note: these three Twitter accounts were discontinued in 2025 and all social media presence has since moved to Bluesky); (3) email communications within the authors' home institutions to reach the internal academic community (e.g. via the School of Medicine at Cardiff University); (4) printed posters displayed at the authors' home institutions; and (5) external email communications circulated by collaborators and partners to their professional networks (e.g. mailing lists of the British Society for Immunology). Completed questionnaires were accepted from anyone who was active or had previously worked or studied in higher education, with no restrictions according to any of the demographics collected.

### Interviews

Questionnaire respondents were able to indicate if they were interested in participating in a semi‐structured follow‐up interview to explore their opinions and views in greater depth. To avoid bias, seven individuals were selected randomly from the total sample of 99 respondents. Interviewees were given an information pack detailing the nature of the interview and the overarching aims of the project. Upon informed consent, semistructured interviews were conducted on a one‐to‐one basis over a Zoom video call with restricted access (Supplementary table 2). Interviews were recorded and stored on a secure server in accordance with local data management guidelines. A full transcript and a summary of key interview outcomes were provided to each interviewee in a debrief document, requiring signed approval and agreement before analysis.

### Analysis

For data‐handling purposes, all respondents were assigned a unique study participation (SP) number. Raw data were processed and analyzed using Microsoft Excel software. Chi‐squared analysis and Kruskal–Wallis tests were used to determine significant differences between experimental parameters, using Minitab Statistical Software and GraphPad Prism. Qualitative content analysis was performed on the interview transcripts to understand perceptions, barriers and facilitators to public engagement.^28^ A coding framework was inductively derived from data; coded transcripts were analyzed to identify patterns, relationships and themes and interpreted in relation to the study's aims.

## AUTHOR CONTRIBUTION

**Chioma M. Ogbukagu:** Investigation, Methodology, Data curation, Formal analysis; Writing—review and editing. **Matthias Eberl:** Conceptualization, Methodology, Formal analysis, Supervision, Visualization, Writing—original draft; Writing—review and editing. **Natalie Joseph‐Williams:** Methodology, Visualization, Writing—review and editing. **Sarah Hatch:** Methodology, Writing—review and editing. **Jonathan M. Tyrrell:** Conceptualization, Project administration, Methodology, Data curation, Formal analysis, Supervision, Writing—original draft, Writing—review and editing.

## CONFLICT OF INTEREST

The authors declare that they have no conflict of interest.

## Supporting information


Supplementary table 1

Supplementary table 2

Supplementary table 3

Supplementary table 4

Supplementary table 5

Supplementary table 6

## Data Availability

The data that support the findings of this study are available from the corresponding author upon reasonable request.

## References

[1] Eberl M , Joseph‐Williams N , Nollett C , Fitzgibbon J , Hatch S . Overcoming the disconnect between scientific research and the public. Immunol Cell Biol 2023; 101: 590–597. 37227221 10.1111/imcb.12657

[2] Allum N , Allansdottir A , Gaskell G , *et al*. Religion and the public ethics of stem‐cell research: attitudes in Europe, Canada and the United States. PLoS One 2017; 12: e0176274. 28426812 10.1371/journal.pone.0176274 PMC5398703

[3] Ittefaq M , Tien Vu H , Zain A , Ramazan T , Kreps GL . Analysis of public opinion polls about COVID‐19 vaccines: theoretical and policy implications for vaccine communication and campaigns to address vaccine hesitancy. Hum Vaccin Immunother 2024; 20: 2437921. 39687950 10.1080/21645515.2024.2437921 PMC11654708

[4] Emery K , Dhaliwal J , Light R , Eberl M , Cruickshank SM . Enabling vaccine uptake: strategies for the public health sector. Br J Hosp Med 2025; 86: 1–14. 10.12968/hmed.2024.0669 40554438

[5] Wolfendale AW . How to spread science to the public – the way ahead? Phys World 1997; 10: 11–12.

[6] Wolfendale AW . The public understanding of science: the Wolfendale report and other matters. AIP Conf Proc 2008; 972: 570–575.

[7] Chowdhury G , Kooya K , Philipson P . Measuring the impact of research: lessons from the UK research excellence framework 2014. PLoS One 2016; 11: e0156978. 27276219 10.1371/journal.pone.0156978 PMC4898824

[8] McLaughlin JA , Boothroyd LG , Philipson PM . Impact arising from sustained public engagement: a measured increase in learning outcomes. Res All 2018; 2: 244–256.

[9] Rip A . The past and future of RRI. Life Sci Soc Policy 2014; 10: 17. 26573982 10.1186/s40504-014-0017-4 PMC4513037

[10] UK Research and Innovation . Framework for responsible research and innovation. https://www.ukri.org/who-we-are/epsrc/our-policies-and-standards/framework-for-responsible-innovation. Accessed September 8, 2025.

[11] Stilgoe J , Owen R , Macnaghten P . Developing a framework for responsible innovation. Res Policy 2013; 42: 1568–1580.

[12] Reed MS , Duncan S , Manners P , *et al*. A common standard for the evaluation of public engagement with research. Res All 2018; 2: 143–162.

[13] Castro‐Sánchez E , Garelick H , Pérez‐Gracia MT , Aminov R . The role of education in raising awareness towards antimicrobial resistance (AMR). Front Microbiol 2024; 15: 1444502. 38989026 10.3389/fmicb.2024.1444502 PMC11233767

[14] Greenhalgh T , Costello A , Cruickshank S , *et al*. Independent SAGE as an example of effective public dialogue on scientific research. Nat Protoc 2025; 20: 1103–1113. 39668237 10.1038/s41596-024-01089-6

[15] World Health Organisation (WHO) . Global action plan on antimicrobial resistance. 2016. https://www.who.int/publications/i/item/9789241509763. Accessed September 8, 2025.

[16] World Health Organisation (WHO) . Implementing the global action plan on antimicrobial resistance: first quadripartite biennial report. 2023. https://www.who.int/publications/i/item/9789240074668. Accessed September 8, 2025.

[17] UK Government . UK 20‐year vision for antimicrobial resistance. 2019. https://www.gov.uk/government/publications/uk-20-year-vision-for-antimicrobial-resistance. Accessed September 8, 2025.

[18] Mochizuki M , Bryan A . Climate change education in the context of education for sustainable development: rationale and principles. J Educ Sustain Dev 2015; 9: 4–26.

[19] Eberl M , Cruickshank SM . A culture shift to support public involvement and engagement in research. J Exp Med 2024; 221: e20240268. 38748084 10.1084/jem.20240268 PMC11096847

[20] Dunleavy K , Noble M , Andrews H . The emergence of the publicly engaged research manager. Res All 2019; 3: 105–124.

[21] Tyrrell JM , Conlon C , Aboklaish AF , *et al*. Superbugs: raising public awareness of antimicrobial resistance through a pop‐up science shop. Res All 2022; 6: 1–21.

[22] Tarín‐Pelló A , Marco‐Crespo E , Suay‐García B , Galiana‐Roselló C , Bueso‐Bordils JI , Pérez‐Gracia MT . Innovative gamification and outreach tools to raise awareness about antimicrobial resistance. Front Microbiol 2023; 13: 977319. 10.3389/fmicb.2022.977319 PMC9520167 36187952

[23] Hatch S , Edwards K , Watson A , Matthews J . The life sciences challenge: delivering an all‐Wales bilingual inter‐school competition for over 10 years and throughout a global pandemic. Res All 2024; 8: 10.

[24] Püttmann V , Ruhose J , Thomsen SL . Academics' attitudes toward engaging in public discussions: experimental evidence on the impact of engagement conditions. Res High Educ 2022; 64: 765–788. 10.1007/s11162-022-09725-4 PMC9734336 36530490

[25] Besley JC , Nisbet M . How scientists view the public, the media and the political process. Public Underst Sci 2013; 22: 644–659. 23885050 10.1177/0963662511418743

[26] Nollett C , Eberl M , Fitzgibbon J , Joseph‐Williams N , Hatch S . Public involvement and engagement in scientific research and higher education: the only way is ethics? Res Involv Engagem 2024; 10: 50. 38822417 10.1186/s40900-024-00587-x PMC11140937

[27] Suri S , Harrison SL , Bevin‐Nicholls A , *et al*. Patient and public involvement and engagement: do we need an ‘ethical anchor’? Res Involv Engagem 2024; 10: 113. 39482787 10.1186/s40900-024-00624-9 PMC11526663

[28] Schreier M . Qualitative Content Analysis in Practice. London: SAGE Publications; 2012.

[29] Tyrrell JM , Hatch S , Flanagan M , *et al*. Superbugs online: co‐production of an educational website to increase public understanding of the microbial world in, on, and around us. Front Microbiol 2024; 15: 1340350. 38384264 10.3389/fmicb.2024.1340350 PMC10879632

[30] Biermann K , Banse L , Taddicken M . “It's mostly a one‐way street, to be honest”: the subjective relevance of public engagement in the science communication of professional university communicators. J Sci Commun 2025; 24: A03.

[31] Besley JC , Dudo A , Yuan S , Lawrence F . Understanding scientists' willingness to engage. Sci Commun 2018; 40: 559–590.

[32] Woitowich NC , Hunt GC , Muhammad LN , Garbarino J . Assessing motivations and barriers to science outreach within academic science research settings: a mixed‐methods survey. Front Commun 2022; 7: 907762.

[33] Tran Dong Thai H , Van Thuy Qui H , Vu Duy T , Fisher J , Chambers M . A study on biomedical researchers' perspectives on public engagement in Southeast Asia. Wellcome Open Res 2023; 8: 202. 37766854 10.12688/wellcomeopenres.19040.2 PMC10521067

[34] Cerrato S , Daelli V , Pertot H , Puccioni O . The public‐engaged scientists: motivations, enablers and barriers. Res All 2018; 2: 313–322.

[35] Rouzer SK , Kalinowski LM , Kaseda ET . The importance of promoting scientific advocacy & outreach for trainees. Neuropsychopharmacology 2023; 48: 713–715. 36631560 10.1038/s41386-023-01530-6 PMC10066368

[36] Andrews E , Weaver A , Hanley D , Shamatha J , Melton G . Scientists and public outreach: participation, motivations, and impediments. J Geosci Educ 2005; 53: 281–293.

[37] Ecklund EH , James SA , Lincoln AE . How academic biologists and physicists view science outreach. PLoS One 2012; 7: e36240. 22590526 10.1371/journal.pone.0036240 PMC3348938

[38] Wilkinson C , Milani E , Ridgway A , Weitkamp E . Roles, incentives, training and audiences for science communication: perspectives from female science communicators. J Sci Commun 2022; 21: A04.

[39] Harris R , Mate‐Sanchez‐Val M , Ruiz Marín M . Gender disparities in promotions and exiting in UK Russell Group universities. Appl Econ 2024; 57: 4441–4457.

[40] Anzivino M . Is public engagement gendered? An analytical proposal using some evidence from Italy. Public Underst Sci 2021; 30: 827–840. 33860694 10.1177/09636625211002060

[41] Lawson C , Salter A . The reverse engagement gap: gender differences in external engagement among UK academics. Stud High Educ 2023; 48: 695–706.

[42] Jonker H , Vanlee F , Ysebaert W . Societal impact of university research in the written press: media attention in the context of SIUR and the open science agenda among social scientists in Flanders, Belgium. Scientometrics 2022; 127: 7289–7306. 35502440 10.1007/s11192-022-04374-x PMC9045683

[43] Ross K , Padovani C . Gender Equality and the Media: A Challenge for Europe. London: Routledge; 2017.

[44] Staniszewska S , Brett J , Simera I , *et al*. GRIPP2 reporting checklists: tools to improve reporting of patient and public involvement in research. BMJ 2017; 358: j3453. 28768629 10.1136/bmj.j3453 PMC5539518

[45] Vinnicombe S , Bianchim MS , Noyes J . A review of reviews exploring patient and public involvement in population health research and development of tools containing best practice guidance. BMC Public Health 2023; 23: 1271. 37391764 10.1186/s12889-023-15937-9 PMC10311710

[46] Aiyegbusi OL , McMullan C , Hughes SE , *et al*. Considerations for patient and public involvement and engagement in health research. Nat Med 2023; 29: 1922–1929. 37474660 10.1038/s41591-023-02445-x

[47] Biddle MSY , Gibson A , Evans D . Attitudes and approaches to patient and public involvement across Europe: a systematic review. Health Soc Care Community 2021; 29: 18–27. 32705752 10.1111/hsc.13111

[48] Lang I , King A , Jenkins G , Boddy K , Khan Z , Liabo K . How common is patient and public involvement (PPI)? Cross‐sectional analysis of frequency of PPI reporting in health research papers and associations with methods, funding sources and other factors. BMJ Open 2022; 12: e063356. 10.1136/bmjopen-2022-063356 PMC9131100 35613748

[49] Mocanu M , Bibiri AD , Rusu VD , Moroșanu A , Gabriel Bejan J . Enhancing civic engagement with science: a comparative approach across European regions. Scientometrics 2025; 130: 447–468.

[50] Varda C , Iordanou K , Antoniou J , *et al*. The role of stewards of trust in facilitating trust in science: a multistakeholder view. J Acad Ethics 2025; 23: 463–483.

[51] Stilgoe J , Lock SJ , Wilsdon J . Why should we promote public engagement with science? Public Underst Sci 2014; 23: 4–15. 24434705 10.1177/0963662513518154 PMC5753839

[52] Burchell K , Sheppard C , Chambers J . A ‘work in progress’? UK researchers and participation in public engagement. Res for All 2017; 1: 198–224.

[53] Grand A , Davies G , Holliman R , Adams A . Mapping public engagement with research in a UK University. PLoS One 2015; 10: e0121874. 25837803 10.1371/journal.pone.0121874 PMC4383503

[54] Kimbrell E , Philippe G , Longshore MC . Scientific institutions should support inclusive engagement: reflections on the AAAS Center for public engagement approach. Front Commun 2022; 6: 787349.

[55] Boon W , de Haan J , Duisterwinkel C , *et al*. Meaningful public engagement in the context of open science: reflections from early and mid‐career academics. Res All 2022; 6: 23.

[56] Swords CM , Porter JS , Hawkins AJ , *et al*. Science communication training imparts confidence and influences public engagement activity. J Microbiol Biol Educ 2023; 24: e00037‐23. 37614888 10.1128/jmbe.00037-23 PMC10443307

[57] Patson ND , Wagner L . Building a more engaged scientist form the bottom up: the impact of public engagement training on undergraduate students. PLoS One 2024; 19: e0302671. 38687727 10.1371/journal.pone.0302671 PMC11060579

[58] Tyrrell JM , Ayanikkad HU , Nalleppillil‐Gopakumar V , *et al*. Combining postgraduate research training, public engagement, and primary school science education‐a superbugs master (MSc) class. Front Microbiol 2024; 15: 1380045. 38881662 10.3389/fmicb.2024.1380045 PMC11176509

[59] Broomfield K , Craig C , Smith S , Jones G , Judge S , Sage K . Creativity in public involvement: supporting authentic collaboration and inclusive research with seldom heard voices. Res Involv Engagem 2021; 7: 17. 33731228 10.1186/s40900-021-00260-7 PMC7968302

[60] Reed MS , Duncan S , Manners P , *et al*. A common standard for the evaluation of public engagement with research. Res for All 2018; 2: 143–162.

[61] Yuan M , Lin H , Wu H , Yu M , Tu J , Lu Y . Community engagement in public health: a bibliometric mapping of global research. Arch Public Health 2021; 79: 6. 33436063 10.1186/s13690-021-00525-3 PMC7801880

[62] Phogat P , Rab S , Wan M . Science communication in the digital age: trends, gaps and interdisciplinary opportunities. Inf Serv Use 2025; 45: 148–163.

