# Supplementary material for: Benefits of public engagement in research and barriers to participation: a UK‐based survey of academic scientists and support staff including international respondents
Source: Immunol Cell Biol. 2026 Jan 9;104(3):192–207. doi: 10.1111/imcb.70079 (PMC12972233; doi:10.1111/imcb.70079)
Supplement: Supplementary file 1 — Supplementary table 1 [file IMCB-104-192-s003.pdf]

Supplemental Table S1

Questionnaire: Attitudes and Barriers to Public Engagement Participation

<u>Section A: Personal Demographics of Respondents</u>

**Question 1**: Country of Origin?
Open Question
**Question 2**: Current country of residence?
Open Question
**Question 3**: Gender
Male
Female
Prefer not to say
Other (Please Specify)
**Question 4**: Age
<21 years
21 – 30 years
31 – 40 years
41 – 50 years
51 – 60 years
61 – 70 years
≥70
**Question 5**: Religion
Christianity
Jewish
Hindu
None
Prefer not to say
Other (please specify)
**Question 6**: Academic Discipline
Open question
**Question 7**: Please state your current job title
Open question
**Question 8**: In which language do/did you receive the majority of your education?
Open question
**Question 9**: Years of experience in academia
≤5 years
6 – 10 years
11 – 15 years
>15 years
**Question 10**: Years of experience in public engagement
≤5 years
6 – 10 years
11 – 15 years
>15 years

Section B: Participation in Public Engagement Initiatives

Question 11: Have you participated in any public engagement activities?

Yes

No

Question 12: If yes to Q11, please specify the type(s) of public engagement activities you were involved in?

Public talks/lectures

Community workshops/seminars

Media interviews and/or articles

School outreach events

Other (Please specify)

Question 13: What was your role in the event(s)?

Lead organiser

Part of the organising team

Volunteer helping

Question 14: Why do you participate in public engagement activities?

Open question

Question 15: In your opinion, are there any benefits from participating in public engagement activities?

Yes

No

Question 16: If Yes to Q15, kindly state the benefits.

Open question

Question 17: Which scientific area do you work/engage the public?

Biomedical sciences (Microbiology/immunology/cell pathology etc)

Medicine/Healthcare Sciences

Psychology/Social Sciences

Biosciences/Natural Sciences

Mathematics/Physics

Other

Question 18: In your opinion what benefit does participating in public engagement initiatives bring?

Increased awareness and understanding among the public

Increased/new skills

Public feeling empowered to act on scientific evidence

Opportunity to build trust in scientific community

Opportunity to improve communication skills

Opportunity to develop project management/leadership skills

Other (please specify)

Question 19: How do you perceive the roles of public engagement initiatives on increasing positive change among the general public?

Low/no impact

Moderate impact

High/positive impact

Very high impact

Question 20: In your opinion, to what extent can quality engagement initiatives contribute to promoting positive change in behaviour?

Not at all

To a small extent

To a large extent

Completely

Question 21: In your experience, have you observed any positive outcomes resulting from public engagement initiatives that you have been involved in?

Yes

No

Question 22: If Yes to Q21, kindly describe your experience

Open question

Question 23: What role do you think academic institutions should play in supporting and promoting participating in public engagement initiatives?

Providing expertise & knowledge

Facilitating collaboration with other stakeholders

Funding support for public engagement initiatives

Creating a supportive culture in which to conduct public engagement activities

Providing adequate training for participation

'Fit for purpose' infrastructure to facilitate collaboration on public engagement

Other (Please specify)

Question 24: Are there any specific challenges or limitations you perceive in the effectiveness of public engagement activities?

Yes

No

Not sure

Question 25: If Yes to Q24, kindly explain the challenges experienced

Open question

Question 26: How do you think public engagement initiatives can be improved or enhanced?

Increasing funding for initiatives

Enhancing collaboration between academia and other stakeholders

Utilising innovative communication strategies

Access to public engagement expertise

Establishment of national engagement initiatives that institutes/academics could engage with

Allocated/ring fenced time within workload models

Other (Please specify)

Section C: Examining academic level of public engagement activities

Question 27: In which country/countries have you participated in public engagement activities?

Open question

Question 28: In which language have you participated in public engagement activities?

Open question

Question 29: How would you rate the level of your personal participation in public engagement activities?

Very low
Low
Undecided
High
Very high

Question 30: To what extent do you perceive your institutions support and encouragement for public engagement activities?

Not at all
To a small extent
To a large extent
Completely

Question 31: Have you ever received training in public engagement?

Yes
No

Question 32: How effective do you believe the public engagement training have you received was in preparing you for such activities?

Very effective
Effective
Neutral
Ineffective

Question 33: How frequently do you engage in public engagement training?

Weekly
Monthly
Quarterly
Annually
Never

Section D: Identifying barriers to academic involvement in public engagement

Question 34: Have you encountered any barriers or challenges in your academic involvement in public engagement initiatives?

Yes

No

Question 35: If Yes to Q34, please select the primary barrier(s) you have encountered

Lack of institutional support or recognition

Limited funding/resources in public engagement

Time constraints or competing priorities

Finding public engagement opportunities to get involved in

Lack of personal interest in public engagement

Lack of academic outputs related to public engagement

Others (Please specify)

Question 36: How significant do you perceive the barriers you have encountered to hinder your academic involvement in public engagement?

Not at all

To a small extent

To a large extent

Completely

Question 37: Do you believe addressing these barriers could enhance academic involvement in public engagement?

Yes

No

Question 38: In your opinion, what strategies could help overcome the barriers to academic involvement in public engagement

Increased institutional support and resources

Dedicated funding for public engagement initiatives

Flexibility in academic workload to accommodate public engagement activities

Creation of networking and collaboration opportunities

Other (Please specify)
