# Supplementary material for: Benefits of public engagement in research and barriers to participation: a UK‐based survey of academic scientists and support staff including international respondents
Source: Immunol Cell Biol. 2026 Jan 9;104(3):192–207. doi: 10.1111/imcb.70079 (PMC12972233; doi:10.1111/imcb.70079)
Supplement: Supplementary file 2 — Supplementary table 2 [file IMCB-104-192-s001.pdf]

Supplemental Table S2

Oral interview guide

1. How do you view the role of public engagement in addressing societal issues?
2. How effective do you think public engagement initiatives are in raising public awareness?
3. In your experience, which public engagement methods are most effective at promoting behavioural change?
4. How do you assess the impact of your public engagement activities?
5. What changes in society behaviours have you observed as a result of your involvement in public engagement activities?
6. Are there any institutional barriers that hinder your participation in public engagement activities?
7. What strategies have you found effective in overcoming these barriers?
8. What kind of support would enhance your ability to participate in public engagement activities?
9. Do you feel that there is adequate recognition and positive impact of academics participating in public engagement initiatives?
10. Can you suggest any innovative approaches to enhance participation and perceived values in public engagement?
