# Supplementary material for: Benefits of public engagement in research and barriers to participation: a UK‐based survey of academic scientists and support staff including international respondents
Source: Immunol Cell Biol. 2026 Jan 9;104(3):192–207. doi: 10.1111/imcb.70079 (PMC12972233; doi:10.1111/imcb.70079)
Supplement: Supplementary file 3 — Supplementary table 3 [file IMCB-104-192-s005.pdf]

Supplemental Table S3

Responses to question Q14 of the questionnaire:

Q14*: "Why do you participate in public engagement activities?"*

Answers to Q14
Because it is my obligation to enlighten my audience on my products specific
Personally rewarding experience and useful for grant applications.
I think it's a vital part of being a scientist - communicating your science to a lay audience. I also enjoy it
For two reasons. 1) Linguistics has a reputation for being hard and abstruse, but in fact my kind of linguistics (sociolinguistics) is about people's relationship(s) with their language(s), so it is something that EVERYONE knows about and can contribute to, even if they have never taken a linguistics class in their lives. Just by the fact of speaking a language, you know a lot about it, socially. So it's fun to show people that, and to see them realise it. 2) I see it as my duty to give back to the public, who pay for a lot of my work through taxes, so I try to do as many public-engagement activities as I can.
A key part of my research. Need to engage with the general public and specific groups e.g. policymakers
Improving my communication skills to make sure someone from most backgrounds understand my research; genuinely enjoy working with the public
Because I believe communicating science is an important part of research, and I see the endless value in it.
Communicating science and knowledge, esp. to children and young people. Insights into research from discussions with members of the public
I enjoy doing it and think it's important to communicate science!
To create awareness about cervical cancer
I enjoy inspiring young people about science. I also enjoy teaching members of the public about research and giving them the opportunity to ask questions/engage with research.
To enlighten the public
To learn
To improve health research, awareness about various health research projects, and improve public understanding of processes in health research - as well as to improve academic and medical funding applications (so there is nothing about individuals, without the input and steering from those individuals).
Fun, important to communicate research with the public especially when it is publicly funded, CV
I participate in public engagement to improve the real-world applicability of my research, improve the quality of my research design, and help disseminate my findings.
I believe it's vital to include and support the Public in all research work we do, especially a diverse group of members of the public, to capture the Public voice and share the transfer of knowledge to learn from each other. It should always be two way meaningful engagement
Mainly because I'm asked to.
Mostly because I enjoy it (same reason I do science), and find it rewarding (same reason I do science)
I enjoy them, I know the public value them, and it's great for students and post docs to meet and talk to people who are impacted by their research
Responsibility, job, can't do good research without all those who hold knowledge
Research impact
I believe sharing our research with general public is part of the role of a researcher. I want my research to be of benefit to the wider population. This requires disseminating and involving the public in our work.
The organisation is part of my job role

It helps to know the need of the community and the best way to address them.
Important to communicate relevant research, keep in touch with those outside academic/science bubble
To share findings with the public, to seek their input into research, to inspire the next generation of scientists
Sharing science should be genuinely democratic, in the sense that it is for and accessible to everyone.
Part of the role of an academic
Public science literacy helps people make informed choices.
There are many reasons, not in order of importance: (1) In think it is important for scientists to communicate science and the nuances of science to the public - to help the public to understand that not all answers are know, there are grey areas and caveats (2) to demonstrate that scientists are real people (3) to help children especially understand that we are all scientists and that curiosity is important and should be encouraged (4) to bring science to life for people - to take science out of the classroom/book/lab (5) to give the opportunity for open discussion between 'scientists' and the public (6) because it is fun - I like talking and engaging with people and learning from them as well! (7) to give the opportunity for people who may not have access to/have completed an education to talk with 'scientists' (8) to break the stereotype of a scientist - as a small female I like to show that science isn't just for those people who look like Einstein/come from a privileged background
It's an expectation / obligation as part of my job but the reason I do this job is because I really enjoy it too. I like sharing my enthusiasm for science and I think people enjoy our activities and have some fun while learning something new.
Understanding science is important for a full comprehension of the world, and of public policy. I want to advance this comprehension in people, for their own enrichment and for a fuller societal engagement of people in matters that impact on their welfare.
Expected part of an academic role
Two way engagement of between scientists and the public is critical to the understanding and reinvigoration of research
To pass scientific information as a job requirement
I enjoy it. I see the benefits of doing it. It gives back to my community.
So important to involve the public in research and science so that they can understand more what we do in the university and ultimately they should help shape the research we do and ground it in reality-after all Universities get a lot of public funding
I think it's important and informs my research
My role, but also because of the major impact that these activities have on individuals, groups, communities
I enjoy enthusing people about science
Passionately believe in its value and important that public can know about how research money is used
To ensure research reaches the people affected by it
There isn't enough room or time to explain but broadly its unethical not to do so
To disseminate my work to as wide an audience as possible. To learn from as many views as possible.
It is essential if research is to make a difference to society in my view
To share my enthusiasm for science and its vital importance to humanity
To help encourage interest and understanding of the latest research and how it relates to them.
To raise the awareness of research and increase its societal impact
Part of my role
(1) a variation from day-to-day academia, it provides interest (2) university requirements to show evidence of 'outreach' activities for promotion etc.
To advance public knowledge of, contribution to and understanding of research
Passion for community service
It's fun and like to help engage the younger generation in science

To help the public understand what we do as scientists and help them understand cancer a little better
To make science more accessible to everyone and to attract their attention.
Mostly because it’s part of the job, to meet funders expectations and because its helps build a projects impact case. I do enjoy public engagement too, but the previously listed reasons are probably the more genuine reasons.
It's important to share publicly funded research with the public. Getting students involved in research at an earlier stage.
We need to raise public awareness of the importance of science
It's part of my job!
I love meeting people and feel it's really important to engage with the public to not just talk about our research but to give them opportunities to shape our research in future.
It might be helpful for recruiting students. Also, I want more people to understand science.
part of my work duties
Career growth
Ultimately our research is meant to serve the public - We can't achieve this if we do not engage with the public
To build my confidence in public speaking
to contribute to public health education
It is important to promote public's understanding and opinion of research since the vast majority of research is publicly funded.
To create awareness, educate and help the public
For continuous professional development
Service to humanity.
To know people's knowledge. also a passion
To help out
It is both part of my role and my passion to make research accessible to the wider public.
I enjoy explaining my research and want more of the public to have access to current research
Encourage young people to explore science and considerer future careers in scientific research
Because my boss asked me, for my Mum's school
Work in a role where it’s part of the strategy
As a Parkinson's researcher, it is important to me to meet and engage with the people my research intends to serve. It gives me motivation as a researcher to see who my research will impact.
Important to ensure appropriate public representation in research, and gain their ideas and perspectives within engagement activities
Fun
Because I wish I’d been able to meet more scientists and had better careers advice when I was at school
Personal fulfilment and wanting to "send the ladder back down".
Bridge the gap between the forefront of research and everyday people, increase understanding of general research methods
