# Supplementary material for: Benefits of public engagement in research and barriers to participation: a UK‐based survey of academic scientists and support staff including international respondents
Source: Immunol Cell Biol. 2026 Jan 9;104(3):192–207. doi: 10.1111/imcb.70079 (PMC12972233; doi:10.1111/imcb.70079)
Supplement: Supplementary file 4 — Supplementary table 4 [file IMCB-104-192-s006.pdf]

Supplemental Table S4

Responses to questions Q15 and Q16 of the questionnaire:

Q15*: "In your opinion are there any benefits from participating in the public engagement activities?"* — Q16*: "Kindly state the benefits."*

Q15	Q16
Yes	As an attendee...? or as an academic...? For academics it ensures their work is relevant and also keeps that connection between their work and the people it benefits. It's also a lot of fun and a break from the day to day.
Yes	Creating awareness and educating the public
Yes	Better able to communicate your research, often working in multidisciplinary groups, develops different skills, and it's fun
Yes	Giving members of the public a better understanding of the methods and working of health research, giving members of the public an opportunity to improve the aims and design of health research, optimising the use of public money.
Yes	Develops soft skills re describing research to a lay audience. Brings new perspectives.
Yes	Feedback and alternative perspectives on our research from people with first hand experience.
Yes	Develop communication skills, how to speak about your work to a lay audience. Could open up young people to things they hadn't heard about before, get them interested from a young age, inspire young people and encourage them to be more ambitious
Yes	Networking, communication skills improvement
Yes	A mix of altruistic and selfish benefits. I like doing it, it's fun, you get to hear other people's perspective on your topic, I enjoy them 'getting' something I've explained, I'm funded by the UKRI and so am able to satisfy grant requirements by doing it, I think it's important and helpful for scientists to make their knowledge available to those who want it (I'd like to see it other disciplines too), it also pays my bills :) You never learn a topic as deeply as when you have to explain it to someone who doesn't share your scientific background!
Yes	You need the community or the public for your initiative to be felt or appreciated.
Yes	By sharing knowledge and you shift power, it gives people a sense of pride to engage. You can often help people in more ways that you realise.
Yes	Confidence and exposure
Yes	Sense of fulfilment and the positive impact on the community
Yes	Improves understanding of immunology in the general public
Yes	Improves my understanding of the reception of research ideas by people the research will ultimately affect
Yes	To learn more
Yes	Meet new people, learn something new from them
Yes	To try and instil a questioning behaviour in people so they don't just believe the press
Yes	Fun, careers benefits, gain leadership skills, gain communication skills.
Yes	First, for the reasons stated in my answer to question 14. Second, since I enjoy teaching and engaging with people interested to learn about what I have to offer.
Yes	improved research impact and methods
Yes	It's rewarding and fun

Yes	Gain confidence. Gain understanding of your own work. It can spark ideas for research. Feels good. raises profile.
No	It improves my public speaking skill
Yes	Improve communication skills
Yes	Serving, Leadership and Networking
Yes	too many to list
Yes	It changes public perspectives and help you understand what the public knows
Yes	Improved communication, more sympathetic response to science from the public
Yes	There is a wide range of benefits from helping to develop professionally in terms of developing communication skills; build trust with members of the public; knowing that your work is meeting the needs of society; it is fun and helps inform what you are doing if done well. For members of the public it can be empowering to be engaged and fun to learn about the work going on in universities.
Yes	having a more clear idea of the public opinion
Yes	Better understanding of what it’s like to live with a certain disease
Yes	Making science accessible
Yes	There are too many to mention here. Benefits to publics, benefits to the researchers too. Engage with science, science careers, learning about the science that publics fund through their taxes, researchers gain confidence sharing their science and so many more!
Yes	Personal fulfilment, increased public awareness and public trust in research
Yes	Improvements to research applicability and feasibility, improved dissemination.
Yes	Better understanding of what the masses want and being able to explain better reasons behind new services
Yes	Improves one's ability to communicate science clearly
Yes	Feel good about sharing science, helps me understand my own work from a different perspective when I am explaining it to others
Yes	Hear views of public, share science
Yes	The reasons for doing outreach/public engagement I stated above are also the benefits of public engagement. (1) In think it is important for scientists to communicate science and the nuances of science to the public - to help the public to understand that not all answers are known, there are grey areas and caveats (2) to demonstrate that 'scientists' are real people (3) to help children (especially) understand that we are all scientists and that curiosity is important and should be encouraged (4) to bring science to life for people - to take science out of the classroom/book/lab (5) to give the opportunity for open discussion between 'scientists' and the public (6) because it is fun - I like talking and engaging with people and learning from them as well! (7) to give the opportunity for people who may not have access to/have completed an education to talk with 'scientists' (8) to break the stereotype of a scientist - as a small female I like to show that science isn't just for those people who look like Einstein/come from a privileged background
Yes	Shared experiences/engagement from the public in research create more relevant and accessible research, which has the ability to enact more effective and positive changes for the people who engaged
Yes	Improved scientific communication skills, gets your work publicised, gain new perspectives on your work, grow your network and possibility for cross-disciplinary collaborations
Yes	Increasing the quality, relevance and impact of research. Personal enjoyment and inspiration.
Yes	Lived experience involvement, advancing public knowledge, conducting real life research
Yes	Mutual understanding, potential to improve the relevance of research and therefore more likely to have impact, hold researchers to account for what they do, unlock power in the public's involvement in driving research

Yes	It improved my sales skills and product sales
Yes	1) highlights questions researchers haven't thought of, 2) helps recruit public for research (participation/involvement etc.), 3) builds public confidence and trust in research, 4) helps widen participation, 5) in psychology is especially important to help repair historic abuse of power between researchers and patients
Yes	Feeling of empowerment and that people have contributed
Yes	Educating the public
Yes	Research impact, career progression, funding requirements
Yes	Improve communication skills, improve understanding of benefits of research, improve interest in and literacy in STEM subjects
Yes	Can demonstrate value for money and impact to funders and host institutions, it builds confidence for the researcher, it enhances research proposals and research protocols etc. it builds connections to the community, strengthens relationships with collaborators. I could go on and on!
Yes	Knowledge translation, transparency, trust, open dialogue, shared learning
Yes	understanding, relevance, enjoyment
Yes	For the patient attending they get to see the innovations going on in the field, for me as a researcher, it allows me to engage with patients and recruit new people to my studies
Yes	dialogue across different disciplines and demographics, learning from each other, demystifying science, inspiring next generations
Yes	It democratises science; It helps inform both the participating public and the researchers; It helps tackle misinformation; and many more.
Yes	Research will be directly informed by the public's priorities, more likely to have engagement with your work as people will share the values of it.
Yes	The public and others gaining knowledge of our research and, I hope, valuing what we do. Informing policymakers of research for use, we hope, in forming policies.
Yes	Developing your own understanding of a subject, giving back to the community, supporting the school curriculum, meeting new people, developing presentation and delivery skills
Yes	To make research more relevant to societal needs.
Yes	See Q14
Yes	See above - fun and challenging
Yes	It feels nice to help someone understand something a little better. People ask interesting questions which make you think about things a little differently
Yes	Improve variety of skills: communication, working with non-science partners, gaining insight from different perspective of my research
Yes	Participating in important conversations about how we shape education and training
Yes	see 14
Yes	Better communication skills, new ideas
Yes	Greater public understanding of research and research which better reflects public concerns and priorities
Yes	Fame
Yes	As above- ensures that the public are encouraged to engage with research, and to gain their ideas and perspectives
Yes	
No	For an academic, there are increasing amounts of career benefits, as engagement is more and more important in successive REFs, so engagement contributions that academics make can

	contribute profitably to their CVs and their promotion prospects. For the public, see above. It's fascinating, surely, to learn that you actually do know something about a thing that someone studies in a university, which you may have thought was way out of your area of expertise.
Yes	So many! for the academics and the community including self-confidence, shared understandings, mutual development, sense of purpose, sense of contributing to something bigger than yourself, experience, fun, learning something new...
Yes	Enthusiasm, ideas, new collaborations, better at teaching
Yes	Improve knowledge base of your target audience, increase the trust in science, develop your skills as a scientist.
Yes	Morally rewarding
Yes	Understanding the needs of communities, building meaningful relationships to continue the engagement work, learning from people with lived experience, future proving research, supporting potential collaboration activities with the Public, building research questions with the public, the Public will have more understanding and trust in academic research, potentially more people will take part in research
Yes	Understand the public perception of the subject matter
Yes	Build confidence, Public speaking , communication and presentation skills
Yes	It's an opportunity to state my views
