# Supplementary material for: Benefits of public engagement in research and barriers to participation: a UK‐based survey of academic scientists and support staff including international respondents
Source: Immunol Cell Biol. 2026 Jan 9;104(3):192–207. doi: 10.1111/imcb.70079 (PMC12972233; doi:10.1111/imcb.70079)
Supplement: Supplementary file 5 — Supplementary table 5 [file IMCB-104-192-s004.pdf]

Supplemental Table S5

Responses to questions Q21 and Q22 of the questionnaire:

Q21*: "In your experience, have you observed any positive outcomes resulting from public engagement initiatives that you have been involved in?"* — Q22*: "Kindly explain your experience."*

Q21	Q22
Yes	Increased teacher confidence in teaching geoscience, increased knowledge of stem careers in students, increased confidence in own ability of students
Yes	I can confidently speak in the public without being twnsedt
Yes	Developed an initiative that trained non-physicists to deliver physics related activities to young girls. Was empowering seeing them enjoy the activities and engage with them. Broke down some fears regarding 'science'
Yes	We changed our research protocol as a result of asking patients and public their views - we were able to recruit better to our clinical trial
Yes	Increased awareness of important topics, increased desire to access a scientific career
Yes	inspiring students, changing opinions on biological research, learning from a different discipline
Not sure	those least likely to change behaviour are also least likely to engage
Yes	Enthusiasm for science increased
Yes	I've seen first hand that knowledge is power and when the public is informed with scientific evidence, this has the power to change minds; behaviours etc.
Yes	After our outreach which encouraged and thought communication skills, we got really good reviews on how it has transformed the individual lives.
Yes	Increased sign ups for open days and applications to undergraduate courses. Developing teacher understanding of the different types of engineering and what engineers can do as careers.
Yes	When I participated in community outreach about the need for parents to immunise their children against measles, months later there was a 30% increase in immunizations in that locality.
Yes	Ability to communicate effectively before the public and utilize my skills positively
Yes	Public can form opinions on healthcare issues based on knowledge/evidence rather than hearsay.
Yes	People have wanted to know more about immunology and feel more engaged
Not sure	I'm not sure that my linguistics public engagement has brought much behavioural change, since it's usually under the category of 'interesting' rather than 'useful'. But public engagement from the hard sciences can prompt behavioural change much more easily, especially if the engagement showcases research which demonstrates that a behavioural change will lead to an improvement in people's own lives, or the lives of their loved ones, or for the planet.
Not sure	Hard to know what the positive outcomes might be, aside from informing others. Bigger impacts may be incremental and only be discerned at a later point.
Not sure	I didn't answer yes
Yes	Please see all previous Superbugs work.
Yes	Positive conversations between members of the public (e.g. children talking positively about food/each other), children engaging with science and asking questions.
Yes	As above

Yes	The excitement from the public about learning about their health. People were eager to get themselves tested and inquired more of how to prevent cervical cancer.
Yes	Students enthused about biological subjects
No	No
Yes	Make some participants aware of their medical status
Yes	Good
Yes	Academics delighted with how public involvement has refined their research questions, methodology design, recruitment design, and outcomes and dissemination.
Yes	Public learning new science and an understanding of science being made up of contributions from many hard working and caring individuals (that science/scientists is not an impenetrable monolith), students/volunteers gaining new skills and confidence.
Yes	People have become interested in taking part in research studies. Others have adapted their lifestyles.
Yes	Community voices being heard in research, confidence building, more people interested in the specific research being discussed, idea creation and development
Not sure	The benefits are long-term and intangible - I have no way to know if any efforts I've made have changed society's long-term attitude to science.
Yes	Building confidence in the school pupils we have worked with, increasing scientific knowledge in the teachers we have worked with, building project management skills in the postdoc who worked on the project.
Yes	We co-produced some leaflets with members of the public explaining medical conditions in lay terms
Yes	building collaborations
Not sure	N/a
Yes	Public and patients have been involved in shaping studies I have been involved in. Understanding their perspectives and experience has helped to shape me as a researcher and continues to be a motivator for my work.
Yes	networking at events has brought community groups/charities etc together that may not have previously been aware of one another
Yes	During vaccination for COVID -19 , the engagement with the public made them to get more enlightened about the efficacy and safety of the vaccine.
Not sure	With one-off interactions, follow up is difficult
Yes	As above. I have seen all the things mentioned above.
Yes	Major conservation progress has been made in my region of the world thanks to public engagement with research results
Yes	.
Yes	I've had audience members talk to me about how they've changed and widened their perspectives. They've been excited about the world again, and that is a good thing.
Yes	An example of a positive outcome has been my engagement with primary schools in South Wales. I gathered feedback from over 100 students and this demonstrated that they had a positive experience and learnt something from my visit. I do not expect to change the world, I am realistic in my impact so I was very happy to have given the children a positive experience - in many cases given the schools I visited my interaction with them was a unique experience.
Yes	Difficult to quantify but we do lots of work with schoolkids and their joy is infectious. In previous roles (medical charities) my activities helped increase fundraising. Competence in public engagement in a variety of fields has also led to funding successes for my team. People have also thanked me and colleagues for explaining a topic to them clearly (health-related).

Not sure	Mainly 'no' to Q21, since positive outcomes are unlikely to be immediate while the public engagement, in my own experience, rarely extends beyond the individual event.
Not sure	Too early to tell and very little follow up to know that the event was the reason for the effect, very easy to claim something had an effect but did it really?
Yes	We have seen a change in awareness of scientific information and its understanding put into context
Yes	The public perception of AIDS ten years when you discuss with people and their perception currently.
Yes	In a group discussion, questions were asked that were used to develop a research proposal. Also public participants felt they had a better understanding of the origins of cancer and that helped them.
Yes	Impact on people's behaviour and health
Yes	purposeful targeted engagement is impactful- when it is designed with a community for a need but your question was broad so I gave it a lower score as some engagement is fun but lacks impacts and some is self serving with no impact
Yes	On individuals - changed behaviour, awareness, self confidence, learning; on groups - actioning changes in communities
Not sure	difficult to measure
Yes	Conversations in families about science, young people helping parents and grandparents to understand new scientific concepts, excitement of trying or finding out something new and interesting but would never have thought about.
Not sure	i didn't answer yes
Yes	too many to list
Yes	I do work on political participation and I have seen members of the public change their attitudes and behaviour to political participation following public engagement events. I also do research on climate change and I have seen members of the public change their attitudes and behaviour to climate change following public engagement events.
Yes	Through community engagement I have set up a charity based on partnership working and relationships between different sectors and organisations that uses evidence and new ways of engaging families to tackle the effects of poverty for children
Yes	Turning people who are initially critical of aspects of science into sympathisers
Yes	Increased motivation from researchers to make a difference, improve understanding of the needs of communities/public
Yes	It started a public debate
Yes	Positive feedback from patient support group and members of the public following events and from website
Yes	These kinds of outreach activities are particularly useful for families who homeschool their children, providing additional experience of 'science' outside the home
Not sure	Did not say yes!
No	NA
Not sure	Engagement with younger people can inspire them to build a career in science. Educating about behaviours or risks associated with different activities and actually how this works can cause people to change their behaviours or choices.
Yes	People understood what 'immunotherapy' meant. Kids learned some of the immune cells
Yes	The feedbacks were amazing. The excitement and curiosity in their eyes were worth the efforts.
Yes	Increased public confidence in science, changed career aspirations after engaging with students, public ability to spread the word about science/research findings amongst their communities

Not sure	I am not always sure of the real world impact.
Yes	I'm a member of an historically excluded group, and people have others out afterwards to share that my visibility helped them feel more included.
Yes	In my work we use public engagement to help shape research projects which makes them more relevant and more successful
Yes	It really helped the public feel VALUED and RESPECTED and PART of research.
Yes	I've seen excitement from recipients and increased interest in science.
No	i put no
Not sure	N/a
Yes	Increased awareness of research in a community with low research knowledge. Improved informed consent and enrolment processes for research
Yes	There was significant lifestyle changes from our health talk
Not sure	.
Yes	Positive impact on my CV
Yes	It has help build my communication skills
Yes	I experienced this during a medical outreach on the benefits of blood donation. After the awareness talk, individuals volunteered to donate knowing it could help save lives. awareness,
Yes	People adopted new ways of doing things.
Yes	The public get more enlightened from these public engagements and previous biases are changed.
Yes	I have increase in knowledge and awareness
Yes	Children more open to follow a career in STEM subject (following school outreach programme)
Not sure	NA
Not sure	People were interested in the talks and said it enhanced their understanding
Not sure	n/a
Yes	Interest in science
Not sure	0
Yes	increased understanding of topic
Yes	It helps to build trust and partnerships between patients and researchers, someone I met had gone from feeling very depressed because he had to retire early due to his condition to becoming positive again after he realised his value in participating in studies and the benefits of taking part in novel interventions
Yes	Without public engagement, most studies cannot proceed. Gaining their perspectives supports recommendations and conclusions for journals too.
Yes	Fame
Not sure	Did you mean question 21?
Yes	Public engagement with schools has led to applicants for jobs with us
Not sure	NA
