# Supplementary material for: Benefits of public engagement in research and barriers to participation: a UK‐based survey of academic scientists and support staff including international respondents
Source: Immunol Cell Biol. 2026 Jan 9;104(3):192–207. doi: 10.1111/imcb.70079 (PMC12972233; doi:10.1111/imcb.70079)
Supplement: Supplementary file 6 — Supplementary table 6 [file IMCB-104-192-s002.pdf]

## Supplemental Table S6

**Responses to questions Q24 and Q25 of the questionnaire:**

Q24: *"Are there any specific challenges or limitations you perceive in the effectiveness of public engagement initiatives?"* — Q25: *"Kindly explain the challenges."*

| Q24 | Q25 |
|---|---|
| Yes | Researcher/academic staff time to engage, knowledge of the importance of public engagement, knowledge of what a good public engagement initiative is (e.g. not just a talk at a school) |
| No | No |
| Yes | Assessing the impact of PE so that you can improve activities (to achieve a specific goal / demonstrate impact) often requires skills (qualitative analysis) that the scientists designing/delivering the PE are not strong in. |
| Yes | The mis-information that public are exposed to on a daily basis can be detrimental to the effectiveness of PE - e.g. flat earth belief etc |
| Yes | Not sure if I'm answering the right question but generally the major limitation in academics engaging is still lack of support or importance placed on engagement by senior leaders at my institution |
| Yes | reaching the right audience, having the correct space in which to deliver |
| Yes | Senior academics who firmly believe public engagement means engaging with fellow academics. Because the title professor bestows infallibility, this view is unchallengeable |
| Yes | Lack of support from department of university |
| Yes | Engagement done well takes time. An academic's role is very time intensive and one of the main challenges is carving out dedicated time to do engagement well. |
| Yes | Ability to get to the right audience |
| Yes | Funding for equipment and time to participate. Lack of funding for roles to facilitate engagement and develop relationships with school and community groups. It can be difficult to reach some groups, such as from schools where travel costs etc. cannot be covered. A diverse range of people are needed to participate in public engagement activities so participants are able to relate to the people delivering the activity, although this can lead to a small number of people being overworked. |
| Yes | Funding / Logistics |
| No | Nil |
| Yes | Time pressures and funding constraints. |
| Yes | I have found it hard to engage with the local diverse population. Despite my best efforts in advertising the events the majority of people who turn up are white middle class people. |
| Yes | I think, for a certain section of the public whom engagement initiatives target, there is still a sense of having had enough of experts--so it can be hard to bring them to see how useful and actually enjoyable academic engagement could be |
| Yes | Funding and resources; general lack of PE resources including staff; one more thing for academics to do on top of everything else! |
| Not sure | I didn't answer yes |
| Yes | Lack of interest on behalf of other academic staff, lack of funding, and a perceived lack of value (in terms of academic output) |
| Yes | Funding, and universities do not give value/merit to these activities in the same way as other academic activities (e.g. publications, obtaining funding) |

| | |
|---|---|
| Yes | Limited funding, not always taken seriously as valid use of time in academia |
| Yes | Lack of funding |
| Yes | Lack of support from some line managers as it isn't seen as important |
| No | No |
| No | N/A |
| Yes | Lack of fund and communication |
| Yes | Adequate ongoing involvement requires time and effort on the part of those involving members of the public.  For these longer-involvement opportunities there must be a plan for the end of that relationship, as well as the initiating and running of the process.  Never involve members of the public without giving them the respect of offering to directly share results from any such work, and without following that through to the end.  Tokenism is already a massive issues for - particularly patient - members of the public. |
| Yes | Senior academics/group leaders not seeing the value, seeing it as a waste of time and therefore discouraging/not supporting students/postdocs in participating, departments not providing supported opportunities for junior students to learn and engage, unsuitable facilities, access issues, lack of provision for disabled people. |
| Yes | Finding funding and support to do this effectively. It often feels like an afterthought in some respects. |
| Yes | Funding, other research members not prioritising engaging with the public, time to create materials for the engagement event and to ensure accessibility has been designed into it - takes time and resources we are just rarely have! |
| Yes | It's not necessarily valued - universities make a song and dance about it when it happens, but they make very little effort to actually establish or support it. As academics we rarely have the time to do it well. |
| Yes | The lack of perceived value by colleagues and universities in comparison to other outputs (grants and papers) means that PE activities are low down the list of priorities for people with very busy schedules. |
| Yes | Money and time |
| Yes | funding, time, capacity, |
| Not sure | N/a |
| Yes | Finding the time to get involved can be difficult. |
| Yes | inclusivity can be a challenge - need to ensure all communications are delivered in multiple media eg sound bites, videos, in various languages etc, venues for events need to be easily accessible, carers, facilitators, translators etc need to be welcomed/provided, meeting must be oragnsied at times & places to suit the public, not to suit the organisers. people need to be reimbursed for their time. most importantly people who attend/engage need to be kept in the loop as if they feel they have been 'used' and not re-engaged then they wont feel valued and keep engaging with the project/service. |
| Yes | Due to time constraints, the expected number of  people don't participate. |
| Yes | Funding, workload (researchers as well as me), supervisor support for ECRs |
| Yes | Time and money |
| Yes | Most academics don't value/prioritize public engagement, which means most aren't trained in it (including students). The result is a lot of ineffective and top-down/deficit-model "communication" that doesn't land well (at best) and is harmful at worst. There are also major disincentives within academia impeding/preventing people from doing public engagement at all, and certainly not in the sustained, reciprocal-relationships fashion in which it ought to be done. |
| Yes | Time |

| | |
|---|---|
| Yes | There is very little time to do it; you will almost always have to do it on your own time and uncompensated.  Also, there needs to be media training to help scientists deal with the sound-bite nature of public media. |
| Not sure | With primary schools I found the biggest difficulty was getting a response from the schools in the first place - the schools are very busy places and so do not always have the time to respond to emails. (nb I emailed schools within areas of deprivation using the contact details on their websites, I also followed up emails with phone calls when I had time) |
| Yes | Funding is a perennial problem. I am lucky to be on a grant but when that ends I may find that my work can't continue as it's not really research, not *quite* outreach (though some crossover) and stable pots of funding for public engagement are rare, though there are pots of money for smaller projects. |
| Yes | Monitoring and measuring the effectiveness of public engagement initiatives, given my answer to question 22. |
| Yes | Usually too broad a topic and too wide an audience target to have any lasting effects |
| Yes | There is a lack of understanding of the value brought by public engagement professionals by many academics, universities and funders |
| Yes | Assumptions and bias |
| Yes | Money, and time. |
| Yes | lack of funding and lack of time in work model to ensure quality in engagement activities. Same public members getting involved- lack of diversity. Challenges with efficient remuneration. A change in culture is required to make engagement integral to our research and education in HEI |
| Yes | again too broad to answer well but if activity lacks purpose or reflection to consider how effective then it won't be effective |
| Yes | Infrastructure, culture, perceived importance (v other areas), budgets, time allocations, access to expertise and training (and awareness of need for these) |
| Not sure | not sure |
| Yes | Lack of funding, not recognised by academic institutions as high value work. |
| Yes | lack of funding, institutional priority, and expertise/skills |
| Yes | too long to write here |
| Yes | The challenge is making it inclusive and engaging the hard to reach. |
| Yes | People can be suspicious of the motives of universities. Sometimes a lack of skill in public engagement can produce negative effects. |
| Yes | Funding, time and breaking down barriers |
| Yes | The constant tension researchers find with other priorities and pressures like teaching, winning research funding and other admin and bureaucracy tasks so they can't find the time. a long-standing research culture that does not recognise the importance of public engagement |
| Yes | negative public perception |
| Yes | Engaging with a wide range of people - its the same group of people who participate each time |
| Yes | Members of the public who engage with these initiatives tend to already somewhat knowledgeable and inquisitive about academia. There are some public demographics that are much more difficult to reach with this type of initiative, however beneficial it may be. |
| Yes | Funding and length of project restrictions |
| Not sure | NA |
| Not sure | N/A |
| Yes | Getting money to carry out events |
| Yes | Speaking for Turkey, there are some anti-science groups who want to block these efforts. It was the biggest challenge we faced |

| | |
|---|---|
| Not sure | na |
| Yes | Time is a major challenge. Being able to connect with multiple and diverse audiences when living in a location where physical distance and internet access remains a challenge. |
| Yes | Finding a common language with a broad audience is challenging |
| Yes | Trying to get other people to recognise the impact of it. Lack of awareness. |
| Yes | Part of infrastructure is the professional services staff - currently a huge issue in that talented public involvement and engagement staff get stuck at a grade 5 with no scope for progression. They therefore leave, and the university loses their unique skillset, their knowledge (which is mainly learnt on the job and can't easily be downloaded all in one from anywhere) and most importantly the relationships that are built with the public are lost. |
| Yes | Time and money.  Part of service load, but not as valued as scholarship of discovery. |
| Yes | time |
| Yes | Funding |
| Yes | Lack of institutional (e.g. dedicated time, resources) and funding support for these activities |
| No | None |
| Yes | limited audience |
| Not sure | N/A |
| No | N/A |
| Not sure | Nil |
| Yes | Support, Media and Marketing bias could affect the reach of public engagement initiatives. |
| Yes | finance and lack of participation |
| Yes | Lack of fund |
| Yes | Often, there is neither funding nor workload availability to dedicate to follow-up activities. |
| Not sure | NA |
| Yes | Effectively advertising the event outside of the university sphere |
| Yes | Public have to want to engage, timelines from an idea to actually discovering something that could benefit people are long and public may not be aware of these timelines |
| Yes | I am forced to do it when I am already overworked. I don't want ring-fenced time; I want to do my job. |
| Not sure | 0 |
| Yes | Often not a priority for researchers or seen as a 'one off' event. Useful PE needs to be strategic and crucially evaluated to see if it has had an impact, and to refine for next activity |
| Yes | All public and patient involvement and engagement activities should be done with the public and patients, not to them. Their comfort, interests, accessibility needs, etc should always be paramount when planning any event. They should not be made to feel like an afterthought, if an engagement event is to go ahead it should do so with a lot of thought, time, and effort put into it. Otherwise it will lead to reduced trust from the public and patients. |
| Yes | Concerns that specific PPIE groups, or as they like to be called 'supergroups', who are self-selected members of the public, can be quite rude and dismissive to researchers.  I have heard them describe how they will decline support for studies and grants if they do use technology they do not understand.  There is also a risk of financial embezzlement by PPIE attenders - claiming for carers allowance, transport, accommodation (often for several days before and after engagement activities), with little trail about what is happening. |
| Yes | People |
| Yes | Time to organise plan and participate on top of the day job is hard to find. |

| | |
|---|---|
| Yes | Reward/recognition lacking for academic colleagues, and practically non-existent for professional colleagues outside specific engagement-style roles. |
| Not sure | NA |
